# Vulnerability factors and neuropsychiatric disorders: What could be learned from individual variability in cognitive functions

**DOI:** 10.3389/fpsyg.2022.1019030

**Published:** 2022-12-22

**Authors:** Elena Daprati, Daniele Nico

**Affiliations:** ^1^Dipartimento di Medicina dei Sistemi and CBMS, Università di Roma Tor Vergata, Rome, Italy; ^2^Dipartimento di Psicologia, Università di Roma La Sapienza, Rome, Italy

**Keywords:** individual differences, Anorexia Nervosa, ritualistic behavior, associative learning, body representation, precision psychiatry, neurocognitive markers

## Introduction

Current studies in the domain of cognitive neuroscience are devised to ensure a high signal-to-noise ratio. Measures from numerous participants are averaged to allow for commonalities to emerge, washing out possible differences. Albeit generally a good thing, a “one-size-fits-all” approach has a drawback: it fails to take inter-individual variability into account, a limitation that is no longer tenable given the increasing attention to so-called precision medicine (Schleidgen et al., 2013).

Recently, personalized approaches have become attractive to psychiatry, raising interest for which biomarkers may characterize each patient (Fernandes et al., 2017; Wium-Andersen et al., 2017; Levchenko et al., 2020). Although most research focuses on genetic and biochemical markers, attention has been paid also to the functional organization of the brain, which is deemed to be largely idiosyncratic and possibly “as unique as a fingerprint” (Finn et al., 2015). Interestingly, both functional (Mueller et al., 2013) and structural variability (Hill et al., 2010; Kanai and Rees, 2011) is larger in association compared to primary cortices, an observation that fits well with the ample differences observed in the population for higher-order cognitive functions. In cognitive terms, variability means that although all individuals eventually attain the same goal, they may do so by means of entirely different strategies (Marchette et al., 2011; Miller et al., 2012). Accordingly, information derived from cognitive styles, personality traits, and/or behavioral strategies can provide relevant clues for understanding and characterizing maladaptive behavior.

Here we discuss ritualistic behavior and body-size delusions as examples of how pathological outcomes and healthy cognitive functioning represent the extremes of a continuum. We argue that inter-individual differences in healthy cognitive style can inform on vulnerability traits or endophenotypes for disease and contribute to characterizing each patient's condition. Adapting Tolstòj's famous quote, we thus maintain that each un-healthy brain is un-healthy in its own way.

### Ritualistic behavior

Ritual is a loose term used to describe series of actions that are repeated over time in rigid, stereotyped manner, and whose function and/or meaning goes beyond their immediate appearance. Repetitive behaviors mostly involve the cortical-striatal-thalamic network associated with habits formation (Graybiel, 2008), a pattern largely conserved across evolution, indicating its robust ecological function (Turbott, 1997; Tonna et al., 2019). In all species, collective rituals contribute to maintaining social norms, strengthen emotional bonding, and promote cooperation (Rossano, 2012). In humans, rituals are especially common in childhood, as part of normal development (De Caluw et al., 2020). Although most people grow out of them, forms of repetitive behaviors persist in adults, often triggered by anxiety, which they can alleviate, either by driving attention to the elementary units of the motor act and thus dispelling intrusive thoughts (Boyer and Lienard, 2008), or by restoring control over the situation and reducing uncertainty (Hirsh et al., 2012). Rituals can take the form of superstitions, which are often highly idiosyncratic, leading people to forcibly rely on personal “lucky” objects or behaviors, especially in stressful conditions (Keinan, 2002; Damisch et al., 2010). Tennis champion Rafael Nadal's courtside ritual of carefully lining up several water bottles is emblematic, but similar behaviors are described in most athletes (Dömötör et al., 2016) and a significant part of the healthy population (Muris et al., 1997). The mechanism by which personal superstitions are established is thought to arise from the (unjustified) reinforcement of purely coincidental associations (Beck and Forstmeier, 2007; Daprati et al., 2019), a process akin to the reward-based type of learning supported by basal ganglia activity (Doya, 2000). In psychiatry, “*repetitive behaviors or mental acts that an individual feels driven to perform in response to an obsession or according to rules that must be applied rigidly*” (DSM-5, APA, 2013) are referred to as compulsions. Although typically associated with obsessive-compulsive disorders (OCD), compulsions emerge in a variety of conditions, including autism, addiction, and anorexia. Neuroimaging studies report anomalies in the frontal-striatal-thalamic network of affected individuals, possibly in response to neuroplastic changes occurring over time, which eventually result in hyperactivation of the caudate nucleus, as would be expected by excess habit generation (Gillan et al., 2015; van den Heuvel et al., 2016; Fineberg et al., 2018; Stein et al., 2019).

Summing up, ritualistic behavior spans from ecologically relevant activities (as in collective rituals reinforcing social norms), to relatively innocuous, though possibly intrusive, routines (as in superstitions), to frankly pathological states (as in compulsions). The common thread is the reliance on the mechanisms supporting habit formation. Neurofunctional models of OCD describe an imbalance between the goal-directed and the habit system of action control, which would lead to over-reliance on the latter (Gillan and Robbins, 2014). To a lesser extent, this could be true also for superstitions, which associate with deactivation of frontal areas, possibly signaling reduced cognitive control over behavior (Rao et al., 2014).

### Body-size delusions

Perceiving our body in space is instrumental to all approach/avoidance interactions with the environment (Sirigu et al., 1991; Schwoebel and Coslett, 2005; deVignemont, 2010). Anorexia Nervosa (AN), a severe eating disorder mostly reported in young women (Treasure and Frank, 2015; Dakanalis et al., 2016), seriously affects this perception. As reported in DSM-5 (APA, 2013), a major diagnostic criterion for AN is a “*disturbance in the way in which one's body weight or shape is experienced*,” the other criteria being significantly low weight and intense and persistent fear of becoming fat. In AN body size is generally overestimated (Schneider et al., 2009; Gardner and Brown, 2014; Mölbert et al., 2017; Brown et al., 2021), a belief that is accompanied by affective and behavioral manifestations. Emotionally, this misperception associates with negative attitudes toward the body, which is regarded as a source of distress (Vocks et al., 2007), possibly due to the tendency to make self-esteem dependent on body weight or shape (APA, 2013). Behaviorally, overestimation of bodily space emerges when anorexics are asked to judge on the possible collision with an external probe (Nico et al., 2010) or the ease with which they can pass through an aperture (Guardia et al., 2010; Keizer et al., 2013), indicating that the bias extends to the perceptual-motor level. In cognitive terms, anorexics seem to be unable to reconcile their perceived body size with the real one, failing to properly view themselves from a non-egocentric standpoint (Bora and Köse, 2016; Konstantakopoulos et al., 2020). The operations of mental rotation and visuospatial reasoning involved in these perspective-changes rely on parietal lobe activity (Nico and Daprati, 2009; Gunia et al., 2021) as do the multisensory integration processes required for a coherent and flexible body representation (Berlucchi and Aglioti, 2010; Daprati et al., 2010; Sereno and Huang, 2014). Congruently, signs of parietal dysfunction emerge in AN in both neuroimaging (Gaudio and Quattrocchi, 2012) and behavioral studies (Grunwald et al., 2002; Guardia et al., 2010, 2013; Nico et al., 2010; Keizer et al., 2013).

Overestimation of bodily space is not exclusive to AN. Young and perfectly healthy individuals misjudge their body size, particularly along the width dimension (Casper et al., 1979; Dolan et al., 1987; Urdapilleta et al., 2010; Fuentes et al., 2013; D'Amour and Harris, 2019; Longo, 2022). A primitive coding of body boundaries emerges as early as 18-h after birth (Ronga et al., 2021), testifying that delimiting bodily space clearly serves ecological purposes. In this sense, systematically perceiving oneself as wider than real size may be an asset because it increases the safety margin that protects against threats (Cooke and Graziano, 2003; deVignemont and Iannetti, 2015). If devoid of negative affective values, this mechanism is particularly advantageous considering that the body undergoes ample variations during one's lifetime, due to development (Adolph, 2008) or other physiological changes (e.g., pregnancy, Franchak and Adolph, 2012; D'Amour and Harris, 2019) and overestimation can facilitate perceptual-motor recalibration. The metric distortion is significantly larger in AN (Gardner and Brown, 2014; Mölbert et al., 2017), and deeply affective-laden (Vocks et al., 2007), but—as previously proposed for compulsions—body-size delusions can be represented as the farther end of a continuum originating in healthy behavior.

### The significance of cognitive variability

So far, we provided two among many possible examples (e.g., agoraphobia, Indovina et al., 2019; fibromyalgia, Sarzi-Puttini et al., 2020) whereby a pathological symptom emerges as one extreme of a continuum stemming from basic (and strongly ecologic) cognitive mechanisms. A comprehensive description of cognitive (mal)functioning in neuropsychiatric disorders is still lacking, but systematically exploring variability in the healthy brain could inform on novel, possible susceptibility factors.

For example, detecting and learning associations, a relevant step in habit formation, varies considerably based on personality traits and cognitive style (Kaufman et al., 2010; Stillman et al., 2014; Blanco et al., 2015). Superstitious individuals are more likely to spot and exploit coincidences than non-superstitious ones (Daprati et al., 2019): this adaptive advantage could additionally represent a vulnerability trait toward developing pathological conducts. Unaffected first-degree relatives of OCD individuals can show behavioral anomalies in executive functioning (Cavedini et al., 2010) and structural changes in the fronto-striatal territory, which could similarly constitute a neurocognitive endophenotype for disease (Vaghi et al., 2017). Structure of the parietal cortex, whose relevance to body and space perception is widely known (Berlucchi and Aglioti, 2010; Sereno and Huang, 2014), shows sexual dimorphism (Levine et al., 2016) and differs widely across individuals. Gray matter density and cortical thickness can vary, and anatomical variations translate in differences in performance at tasks of attention switching (Kanai et al., 2010), mental rotation (Koscik et al., 2009) and experience of body ownership (Matuz-Budai et al., 2022), which in turn could make some individuals exceptionally vulnerable to body-size delusions.

In sum, susceptibility factors for pathology may be nested within cognitive variability and—though still poorly explored—should be considered alongside psychosocial and biological markers (Jacobi et al., 2004; Levchenko et al., 2020).

## Conclusion

Recent neuropsychological approaches to psychiatry have underlined the multifactorial origin of mental illnesses, drawing attention to cognitive variables (Wood et al., 2009). Besides permitting a more comprehensive view of disease, exploring cognition provides objective, quantitative measures that can by-pass top-down influences produced by psycho-affective attitudes, an obvious advantage when self-report is affected for example, by denial of illness. The next step forward is feeding information on cognitive *variability* into the newly developing models of disease.

The study of cognitive variability is notoriously laden by methodological and reliability issues (Hedge et al., 2018). Nevertheless, guidelines are rapidly emerging regarding experimental paradigms, populations, and statistical analyses (Mollon et al., 2017; Hedge et al., 2018; Goodhew, 2020), which warrant strong internal reliability, minimize confounds, and allow discriminating between state and trait variables (Goodhew, 2020). Though still scant, information obtained from cognitive variability could thus shed light on vulnerability traits or endophenotypes for disease, contributing to personalizing diagnostic and remediation pathways.

Consider the example of body-size delusions. Describing one's body implies reporting beliefs and/or attitudes related to it *and* applying the visuospatial skills required to view oneself “from the outside” (deVignemont, 2010; Mölbert et al., 2017). While the first aspect is routinely assessed via clinical interviews and scales, the latter is rarely approached, though there are advantages in collecting measures that are quantifiable and less permeable to emotions. Poor perspective-taking abilities can reduce the capacity to take a non-egocentric view about the self, impairing illness awareness and reducing insight on the real state of one's body (Bora and Köse, 2016; Konstantakopoulos et al., 2020). As such, they may both support the symptom *and* constitute a vulnerability trait for disease. Detecting this trait within the healthy population (whereby variability exists; Samuel et al., 2022) can improve screening and diagnostic protocols. Likewise, many remediation programs now employ virtual reality: suitability for these approaches is affected by structural variability in parietal areas (Hosoda et al., 2021). Assessing skills relying on parietal functioning may thus help singling out which individuals will benefit from these therapies. Similar reasoning applies to associative learning: sensitivity to detecting coincidences can belong in an endophenotype for developing ritualistic behavior, as hinted by associations between implicit learning, reward processing and polymorphism for BDNF genes (see Daprati et al., 2019 for a discussion).

Thus, while not implying a causal link between one cognitive style and development of a psychiatric condition, investigating cognitive variability could prove fruitful in characterizing disease and informing on the most probable direction malfunctioning could take, should other factors co-occur.

## Author contributions

ED drafted the paper. All authors discussed the ideas presented in the paper. All authors reviewed and approved the submitted version.
